# Neuroplastin 65 deficiency reduces amyloid plaque formation and cognitive deficits in an Alzheimer’s disease mouse model

**DOI:** 10.3389/fncel.2023.1129773

**Published:** 2023-05-05

**Authors:** Dan-Dan Wu, Jie Cheng, Ya-Ni Zheng, Yu-Tong Liu, Shuang-Xin Hou, Li-Fen Liu, Liang Huang, Qiong-Lan Yuan

**Affiliations:** ^1^Department of Neurology, Shanghai Tongji Hospital, Tongji University School of Medicine, Shanghai, China; ^2^Department of Radiology, University of Nebraska Medical Center, Omaha, NE, United States; ^3^Department of Neurobiology, Shanghai Pudong Hospital, Fudan University, Shanghai, China; ^4^Department of Human Anatomy, Histology and Embryology, Tongji University School of Medicine, Shanghai, China

**Keywords:** neuroplastin 65, Alzheimer’s disease, amyloid plaques, neuroinflammation, 5-hydroxytryptamine receptor 3A subunit, glial activation

## Abstract

**Introduction:**

Alzheimer’s disease (AD) is characterized by increasing cognitive dysfunction, progressive cerebral amyloid beta (Aβ) deposition, and neurofibrillary tangle aggregation. However, the molecular mechanisms of AD pathologies have not been completely understood. As synaptic glycoprotein neuroplastin 65 (NP65) is related with synaptic plasticity and complex molecular events underlying learning and memory, we hypothesized that NP65 would be involved in cognitive dysfunction and Aβ plaque formation of AD. For this purpose, we examined the role of NP65 in the transgenic amyloid precursor protein (APP)/presenilin 1 (PS1) mouse model of AD.

**Methods:**

Neuroplastin 65-knockout (NP65^–/–^) mice crossed with APP/PS1 mice to get the NP65-deficient APP/PS1 mice. In the present study, a separate cohort of NP65-deficient APP/PS1 mice were used. First, the cognitive behaviors of NP65-deficient APP/PS1 mice were assessed. Then, Aβ plaque burden and Aβ levels in NP65-deficient APP/PS1 mice were measured by immunostaining and western blot as well as ELISA. Thirdly, immunostaining and western blot were used to evaluate the glial response and neuroinflammation. Finally, protein levels of 5-hydroxytryptamin (serotonin) receptor 3A and synaptic proteins and neurons were measured.

**Results:**

We found that loss of NP65 alleviated the cognitive deficits of APP/PS1 mice. In addition, Aβ plaque burden and Aβ levels were significantly reduced in NP65-deficient APP/PS1 mice compared with control animals. NP65-loss in APP/PS1 mice resulted in a decrease in glial activation and the levels of pro- and anti-inflammatory cytokines (IL-1β, TNF-α, and IL-4) as well as protective matrix YM-1 and Arg-1, but had no effect on microglial phenotype. Moreover, NP65 deficiency significantly reversed the increase in 5-hydroxytryptamine (serotonin) receptor 3A (Htr3A) expression levels in the hippocampus of APP/PS1 mice.

**Discussion:**

These findings identify a previously unrecognized role of NP65 in cognitive deficits and Aβ formation of APP/PS1 mice, and suggest that NP65 may serve as a potential therapeutic target for AD.

## Background

Alzheimer’s disease (AD) is the main cause of dementia with a population prevalence which increases rapidly. It is clinically characterized by progressive cognitive dysfunction, progressive cerebral deposition of amyloid beta (Aβ) and accumulation of neurofibrillary tangles. So far, the molecular mechanisms underlying cognitive deficits in AD remain still unclear. Neuroplastin 65 (NP65), a member of the cell-adhesion molecule family, is a synaptic glycoprotein which is related with synaptic plasticity and complicated molecular events underlying learning and memory (Ilic et al., 2021). However, whether NP65 is implicated in cognitive dysfunction of AD is still unknown.

Neuroplastin (NP) isoforms are identified as glycoproteins with molecular weight of 65 kDa (NP65) and 55 kDa (NP55), consisting of a short intracellular C-terminal domain, a transmembrane domain, and extracellular Ig modules (Hill et al., 1988; Langnaese et al., 1997; Smalla et al., 2000; Herrera-Molina et al., 2014). Both isoforms are encoded by the same gene (Nptn in rodents and Nptn in humans), and result from alternative splicing of the mRNA, and are distinguished solely by the presence of the Ig1 module specifically in NP65 and absence in NP55 (Langnaese et al., 1997; Smalla et al., 2000). NP55 is widely expressed in various tissues, whereas NP65 is mainly distributed in brain (Smalla et al., 2000; Bernstein et al., 2007; Sakaguchi et al., 2016; Sumardika et al., 2019). In cerebral cortex, NP65 immunoreactivity is primarily present in neuropil regions of layers II, III, and Vb/VI and barrel fields in layer IV of the somatosensory cortex. In hippocampal formation, the prominent NP65 immunoreactivity is detected in surface of granular and pyramidal body cells, the stratum oriens and the stratum radiatum of the CA1 region, and inner molecular layer of the dentate gyrus (DG) (Smalla et al., 2000; Bernstein et al., 2007). Recent study confirmed that NP65 displays similar distributive patterns in human brains compared with rodents (Herrera-Molina et al., 2017). Notably, human entorhinal cortex had a high NP65 expression in pyramidal neurons in the layers II, IV, and V (Herrera-Molina et al., 2017).

Accumulating evidence shows that NP65 is implicated in learning and memory in rodents by mediating activity-dependent synaptic plasticity and neuronal plasticity (Smalla et al., 2000; Empson et al., 2006; Owczarek et al., 2011). Recently, several studies investigated cognitive roles of NP using genetically engineered mice with NP55/65 or NP65 deficiency (Amuti et al., 2016; Bhattacharya et al., 2017; Herrera-Molina et al., 2017; Li et al., 2018). NP-deficient mice (both NP65 and NP55 are deleted in all neurons) or Emx1*^Cre^*:Nptn*^lox/lox^* mice (both NP65 and NP55 are deleted in glutamatergic neurons) showed aberrant swim behavior, indicated by diving and pausing swimming during acquisition phase in Morris water maze (MWM) test, which compromised the evaluation of spatial memory (Bhattacharya et al., 2017; Herrera-Molina et al., 2017). In addition, compared to respective control mice, NP-deficient mice and tamoxifen-induced Pr*^CreERT^*:Nptn^Δ^ *^lox/lox^* mice showed cognitive deficits in shuttle box two-way active avoidance test. In fear conditioning test, NP-deficient mice displayed impaired contextual memory (Bhattacharya et al., 2017). Thus, NP-deficient mice show evident cognitive deficits in associative learning and memory (Bhattacharya et al., 2017). Meanwhile, our previous findings confirmed that NP65 knockout (KO) mice (NP65 is deleted while NP55 is intact) showed enhanced spatial learning and memory measured by MWM test and novel object recognition (NOR) test (Amuti et al., 2016; Li et al., 2018, 2019), which was reproduced by different individuals in our lab. In fear conditioning test, NP65 KO mice showed less freezing in the cue stimulus and a reducing trend of freezing time in the context stimulus (Amuti et al., 2016), indicating cognitive deficits in associative learning and memory, similar to the observations by Bhattacharya et al. (2017). Therefore, NP65 KO mice display differences in spatial and associative learning and memory.

Recently, Ilic et al. (2019) have reported that changes in NP65 expression seem to be related to the course of AD disease. They observed that NP65 was significantly up-regulated in the hippocampus (dentate gyrus, CA2/3 region, and subiculum) with no changes in neuron number or tissue volume in the early phase (≤4 years) of confirmed AD neuropathology. Interestingly, patients experiencing a long duration of AD (5–7 years) showed a reduced expression of NP65 compared to AD patients with a short duration (≤4 years), but had no difference compared with controls (Ilic et al., 2019). Thus, these preliminary results suggest that NP65 might be related with AD pathology.

In light of the possible role of NP65 in the cognitive behaviors and the course of AD disease, we hypothesized that NP65 would be involved in cognitive deficit and Aβ plaque formation of AD. To this end, APP/PS1 mouse (a commonly used model of AD) was used to investigate potential roles of NP65 in AD. APP/PS1 mouse shows pathological cellular and behavioral features of AD, including increasing accumulation of cerebral Aβ plaques, activated glial cells around Aβ plaques and cognitive impairment (Garcia-Alloza et al., 2006; Hammerschmidt et al., 2013; Liu et al., 2020). As APP/PS1 mouse exhibits a significant increase in plaque burden and severely impaired cognitive behaviors at the age of 10 months (Liu et al., 2020), thus, 10-month-old AD mice and age-matched NP65-deficient AD mice were used in present study. Consistent with our expectation, we found that NP65 deficiency reduced amyloid levels and amyloid plaques, and alleviates cognitive deficits in APP/PS1 mice.

## Materials and methods

### Animals

Neuroplastin 65-knockout (NP65^–/–^) mice described in our previous reports (Amuti et al., 2016) and double transgenic APP_*SWE*_ and PS1_Δ__*E*9_ (APP/PS1) mouse of AD model (obtained from Nanjing Biomedical Research Institute of Nanjing University, Nanjing, China) were used. All mice were on the same genetic background (C57BL/6). APP/PS1 mice were crossed with NP65KO mice to obtain NP65^+/–^APP/PS1 mice. We then crossed NP65^+/–^APP/PS1 male mice with NP65^+/–^ female mice to get NP65^+/+^APP/PS1 (AD), NP65^–/–^APP/PS1 (KOAD), NP65^+/–^APP/PS1, NP65^+/–^, and NP65^–/–^(KO) and wild type (WT) littermates. All procedures were in accordance with an animal protocol approved by Tongji University Animal Care Committee. Animals were bred and maintained in a 22 ± 1°C, 40–60% humidity environment with a 12 h light/dark (9:00–21:00) cycle. Food and water were supplied to them *ad libitum*. Animal sufferings and discomforts were minimized during all the procedures.

### Genotyping of the offspring

Offspring genotype of NP65^+/–^APP/PS1 male mice with NP65^+/–^ female mice was identified by polymerase chain reaction (PCR) of tail DNA extracts at the age of 4 weeks using Conway Genomic DNA Extraction Kit (CoWin Biosciences, Jiangsu, China). According to the manufacturer’s protocol, PCR reaction system (50 μl) was composed of primers and 2 μl DNA template and was amplified in a PCR instruments (iQ5, Bio-Rad Laboratories, Hercules, CA, USA). The primers used were as follows: NP65-loxp-tF: 5′-TAGGGAAATGGGAAATCAGT-3′ and NP65-loxp-tR:5′-GGTTCTATTCTGGGCTTGGA-3′; NP65-FRT-tR:5′-CCCATAGGAAAGATCCCTTGTT-3′, and Zmk-2F4: 5′-GCATCGCATTGTCTGAGTAGGTG-3′; PS1: PS1-1644: 5′-AATAGAGAACGGCAGGAGCA-3′ and PS1-1645: 5′-GCCATGAGGGCACTAATCAT-3′; APP: APP-1597: 5′-GCCATGAGGGCACTAATCAT-3′ and APP-1598: 5′-CTTGTAAGTTGGATTCTCATATCCG-3′. The protocol of the PCR was as follows: initial denaturation at 94°C for 2 min, followed by 35 cycles at 94°C for 30 s, and 56°C for 30 s, ended at 72°C for 30 s and 72°C for 2 min. The final products of PCR were separated by agarose gel electrophoresis, and then followed to be photographed with a Gel Imager (Gel Doc XR +, Bio-Rad Laboratories, Hercules, CA, USA).

### Behavioral tests

A cohort of 10-month-old WT, KO, AD, and KOAD mice (*n* = 7–8/genotype) was used to do NOR and MWM tests. NOR test was completed in a box (40 cm × 27 cm × 19 cm, ORT-200A, Techman, Chengdu, China) as previously described (Li et al., 2019). On day 1, mice were allowed to be habituated in the box in absence of objects for 5 min. On day 2, two identical objects were placed in the box and each mouse was allowed to freely explore the objects for 10 min. On day 3, one object was replaced by a novel and unfamiliar object, and the mice were allowed to explore the two objects for 10 min. Exploration of the familiar or the novel object was recorded using a video camera. Exploration was considered when a mouse showed investigative behaviors (sniffing closely or entering an area within 2 cm around the object, heading toward the object). Exploration time was calculated blindly. Recognition index (RI) is the ratio of novel exploration time to total exploration time, and a higher RI means better recognition memory (Oliveira et al., 2010).

After an interval of 3 days, these mice were continued to do MWM test as described in our previous study (Amuti et al., 2016). During the 5 consecutive days of acquisition training, mice were placed facing the wall at one of four quadrants and allowed to find the submerged platform in 60 s and stay on the platform 15 s (four trials per day). On day 6, a probe test was performed after the submerged platform was removed. The mice were placed at the edge of the pool from any of four quadrants and allowed to freely swim for 60 s in search of the removed platform. On day 7, a visible platform test was done, with a visible platform placed above the water surface. The mice were allowed to swim to find the visible platform. The escape latency of mice to locate the hidden platform, swimming pathway, and the times in the target quadrant were automatically recorded by a digital device connected to a computer.

### Tissue preparation

A separate cohort of 10-month-old WT, KO, AD, and KOAD mice (*n* = 3–4/genotype) was intraperitoneally anesthetized with 1% pentobarbital sodium and perfused with 4% paraformaldehyde (PFA). After brains were post-fixed in 4% PFA for 10–16 h, and then placed in 30% sucrose in 0.01 M phosphate buffer for 20–24 h at 4°C. Coronal sections (15 μm thick) were cut using a cryostat microtome (CM 1950, Leica, Heidelberger, Germany) and used for the staining.

### Immunostaining

For immunolabeling, brain slices were blocked with 5% bovine serum albumin (BSA) for 1 h after permeabilized with 0.3% Triton X-100 at room temperature for 10 min. Then the slices were incubated overnight at 4°C with primary antibodies listed in Table 1, including mouse anti-6E10, rabbit anti-Iba-1, and rabbit anti-GFAP. After three times washing by 0.01 M phosphate-buffered solution (PBS), the sections were incubated for 2 h in Cy3-conjugated or fluorescein isothiocyanate (FITC)-conjugated goat anti-mouse or goat anti-rabbit secondary IgG (1:1,000, Jackson ImmunoResearch Laboratories, West Grove, PA, USA), and counterstained with diamidino-phenyl-indole (DAPI, Beyotime Biotechnology, Shanghai, China). For CD68 or Htr3A staining, sections were treated with citrate sodium buffer (pH 6.0) in water bath for 5 min at 95°C to expose the antigen. After washing, sections were incubated with the primary antibodies against Htr3A or CD68 at 4°C overnight, and further incubated for 3 h with biotinylated horse anti-mouse or horse anti-rabbit secondary IgG (1:500, Vector Laboratories, Newark, CA, USA) for 3 h at room temperature in the dark. Finally, sections were incubated for 1 h with ExtrAvidin-Cy3 (1:1,000, Sigma-Aldrich, Louis, MO, USA) at room temperature, and were counterstained with DAPI.

**TABLE 1:** Primary antibodies used in the study.

Primary antibodies	Vendors	Dilution
Mouse anti-interleukin-1beta (IL-1β)	Santa Cruz, Dallas, CA, USA	1:100 immunostaining
		1:500 western blot
Mouse anti-tumor necrosis factor alpha (TNF-α)	Santa Cruz, Dallas, CA, USA	1:100 immunostaining
Mouse anti-chitinase 3-like 3 (YM-1)	Santa Cruz, Dallas, CA, USA	1:100 immunostaining
		1:500 western blot
Mouse anti-arginase1 (Arg-1)	Santa Cruz, Dallas, CA, USA	1:100 immunostaining
Mouse anti-cluster of differentiation (CD36)	Santa Cruz, Dallas, CA, USA	1:100 immunostaining
		1:500 western blot
Mouse anti 5-hydroxytryptamine receptor 3A subunit (Htr3A)	Santa Cruz, Dallas, CA, USA	1:100 immunostaining
		1:500 western blot
Rabbit anti-cluster of differentiation 68 (CD68)	Abcam, Cambridge, UK	1:100 immunostaining
		1:500 western blot
Rabbit anti-glial fibrillary acidic protein (GFAP)	Abcam, Cambridge, UK	1:1,000 immunostaining
		1:500 western blot
Rabbit anti-ionized calcium binding adapter molecule-1 (Iba-1)	Wako, Osaka, Japan	1:500 immunostaining
		1:500 western blot
Mouse anti-beta amyloid 1–16 (6E10)	Santa Cruz, Dallas, CA, USA	1:600 immunostaining
		1:1,000 western blot
Rabbit anti-neuronal nuclei (NeuN)	Abcam, Cambridge, UK	1:500 western blot
Rabbit anti-synaptophysin (SYN)	Abcam, Cambridge, UK	1:500 western blot
Goat anti-PSD-95 (post-synaptic marker post-synaptic density-95)	Abcam, Cambridge, UK	1:500 western blot
Rabbit anti-B-cell lymphoma-2-associated X(BAX)	Proteintech, Chicago, IL, USA	1:500 western blot
Rabbit anti-β-site APP cleaving enzyme 1 (BACE1)	Santa Cruz, Dallas, CA, USA	1:500 western blot
Mouse anti-glyceraldehyde-3-phosphate dehydrogenase (GAPDH)	Beyotime Biotechnology, Shanghai, China	1:000 western blot
Mouse anti β-actin	Beyotime Biotechnology, Shanghai, China	1:1,000 western blot
Goat anti-neuroplastin 65 (NP65)	R&D Systems, Minneapolis, MN, USA	1:500 western blot

To determine whether microglia and astrocytes phagocytose Aβ plaques and express Aβ-binding receptors CD36 as well as inflammatory cytokines, double immunostaining was performed. For double immunostaining of Iba-1/6E10 and GFAP/6E10, sections were simultaneously incubated with these two primary antibodies at 4°C overnight, and then incubated with Cy3-conjugated goat anti-rabbit secondary IgG and FITC-conjugated goat anti-mouse secondary IgG (1:1,000, Jackson ImmunoResearch Laboratories, West Grove, PA, USA) and were counterstained with DAPI. For double immunostaining of CD68/6E10 and Iba-1 or GFAP with IL-1β, TNF-α, IL-4, YM-1, Arg-1, and CD36, the sections were first incubated with these two primary antibodies overnight at 4°C. Brain sections were then incubated with biotinylated horse anti-rabbit or anti-mouse secondary IgG (1:500, Vector Laboratories, Newark, CA, USA) for 3 h in the dark, then incubated with ExtrAvidin-Cy3 (1:1,000, Sigma-Aldrich, MO, USA, Louis, USA) and FITC-conjugated goat anti-mouse IgG or anti-rabbit IgG (1:1,000, Jackson ImmunoResearch Laboratories, West Grove, PA, USA) for 1.5 h. Finally, sections were counterstained with DAPI and mounted.

### Quantification for staining

Mouse brains were cut into 15 μm thick consecutive sections. One brain slice was selected from every six sections and a total of three to four slices of each brain covering the dorsal hippocampus were used for staining and quantification. For cerebral cortex, a total of eight non-overlap images in each side can be captured under 200 × or 400 × magnification. For hippocampus, a total of three non-overlap images in each side can be captured under 200 × or 400 × magnification. Eight images in each side of cerebral cortex were considered to be sufficient to represent the area of cerebral cortex in each section. For more captured images in hippocampus, two sides of hippocampus were used and measured in each section. There has not hitherto been report showing the difference between right and left hemisphere of used mice (Amuti et al., 2016; Li et al., 2019; Liu et al., 2020, 2023). Therefore, bilateral hippocampus and unilateral cerebral cortex were used for immunostaining analysis. For 6E10, GFAP, CD68, and Iba-1 immunostaining, fluorescence was visualized under 40 × (for 6E10) or 100 × (for GFAP, CD68, and Iba-1) magnification in each section using a fluorescence microscope (Olympus, BX51, Tokyo, Japan). The immunopositive area was determined by outlining the boundaries of the fluorescence areas under a user-defined threshold in each field using ImageJ software V1.51 (National Institutes of Health, Bethesda, MD, USA). To perform this, a fluorescent image was first opened in Image J software. Secondly, set pixels to subtract background; Thirdly, convert a fluorescent image to a 8-bit type grayscale image; Fourth, threshold was adjusted to cover all positive areas. Lastly, the positive area and the total area of the image were measured. Percentage of positive area occupied by fluorescence to a total area of this image was defined as the intensity of positive fluorescent signal, indicating expression level of the interested protein.

For double immunostaining analysis of Iba-1 with IL-1β, TNF-α, IL-4, YM-1, Arg-1, and CD36, six images were acquired from the stratum radium of CA1-CA2-CA3 in bilateral hippocampus (three images in each side) and eight images were taken from unilateral cerebral cortex overlying unilateral hippocampus under 200 × magnification using Nikon confocal microscope (Nikon, Melville, NY, USA). During taking pictures, Iba-1 images (FITC-conjugated) were excited at 490 nm and emitted light was captured at 550 nm; the images of IL-1β, TNF-α, IL-4, YM-1, CD36, and Arg-1 (all Cy3-conjugated) were excited at 554 nm and emitted light was captured at 568 nm. The area of each field under 200 × magnification is 0.50 mm^2^ (0.89 mm × 0.555 mm) from a focal plane in brain section (15 μm thick). The percentage of immunopositive area occupied was measured as above in each field under a user-defined threshold by red, green or yellow fluorescence (co-localized analysis with red and green fluorescence in merged images) using ImageJ software V1.51. The intensity of positive fluorescent signal was measured as above.

For analysis of microglial Aβ phagocytosis using Iba-1/6E10 double staining, eight images from the unilateral cerebral cortex were acquired under 400 × magnification. For quantification of the Aβ internalization (intracellular Aβ) in Iba-1(+) microglia, the area of Aβ within microglia [Aβ (+)/Iba-1(+)] was measured using ImageJ software V1.51. The double positive area of Aβ (+)/Iba-1(+) immunoreactivity represents Aβ engulfed by microglia, indicative of intracellular Aβ. In addition, Iba-1(+) microglia within a 20 μm range of Aβ plaques was measured using ImageJ for the RGB fluorescence intensity profile plot as previously reported (Krauthausen et al., 2015).

### Aβ1–40 and Aβ1–42 assay by ELISA

After completed with behavioral tests, the mice (WT, KO, AD, and KOAD, *n* = 3–4/group) were deeply anesthetized with 1% pentobarbital sodium, bilateral cerebral cortex and hippocampus were collected for Aβ1–40 and Aβ1–42 assay. Snap-frozen hippocampus and cortex were homogenized in ice-cold modified PBS using a homogenizer. The homogenates were then extracted in RIPA buffer (25 mM Tris–HCl, pH 7.5, 0.1% sodium dodecyl sulfate (SDS), 1% NP40, 150 mM NaCl, 0.5% NaDOC) and centrifuged at 20,000 × *g* for 10 min at 4°C, the supernatants were collected containing soluble Aβ1–40 and Aβ1–42. The pellets were further dissolved in SDS buffer (containing 25 mM Tris–HCl, pH 7.4) and centrifuged again to collect the supernatants residual insoluble Aβ1–40 and Aβ1–42. After pulsed sonication treatment for 15 s, the protein concentration from RIPA and SDS fraction was determined by BCA Protein Assay Kit (Beyotime Biotechnology, Shanghai, China). Levels of Aβ1–40 and Aβ1–42 in RIPA-soluble and SDS-insoluble fractions were detected using human Aβ1–40 and Aβ1–42 ELISA kits (Mlbio biotechnology, Shanghai, China) according to the manufacturer’s protocol. All samples were analyzed in duplicate.

### Western blotting

A separate cohort of 10-month-old WT, KO, AD, and KOAD (*n* = 3) were sacrificed under anesthesia with 1% pentobarbital sodium. The cerebral cortex and hippocampus were rapidly removed and frozen in liquid nitrogen and then stored at −80°C till further use. In addition, a cohort of 2-month-old WT, KO, AD, and KOAD (*n* = 3) mice were sacrificed as above, and the forebrains were specifically used to confirm that NP65 is deleted in KO and KOAD mice.

Total protein was extracted and concentration determination from the frozen tissues as described above. A total of 15 μg protein samples were loaded per lane, and proteins were separated by electrophoresis and transferred to nitrocellulose membranes. The membranes were incubated with 5% BSA in TBS containing 0.05% Tween-20 for 1 h at room temperature, and then incubated with primary antibodies (Table 1) overnight at 4°C, including rabbit anti-GFAP, rabbit anti-Iba-1, goat anti-PSD-95, rabbit anti-SYN, rabbit anti-Bax, rabbit anti-NeuN, mouse anti-YM-1, mouse anti-CD36, mouse anti-BACE1, mouse anti-6E10, mouse anti Htr3A, and mouse anti-GAPDH or anti-β-actin antibodies. Blots were then washed and further incubated with horseradish peroxidase-conjugated goat anti-rabbit or anti-mouse secondary IgG or donkey anti-goat secondary IgG for 2 h at room temperature. The immunoreactivity was visualized by super Excellent Chemiluminescent Substrate (Beyotime Biotechnology, Shanghai, China) and the images were quantitatively analyzed by ImagJ1.51. The interested protein levels were normalized to GAPDH or β-actin from three independent experiments.

### Statistical analysis

Statistical analyses were carried out with SPSS 22.0 (IBM SPSS, Chicago, IL, USA) for Windows. All data were expressed as mean ± S.E.M. A two-tailed Student’s *t*-test was used to evaluate the differences between two groups. Data for multiple comparisons were analyzed by one-way analysis of variance (ANOVA) followed by a least significant difference test if the data are normal distribution. Otherwise, the Mann-Whitney *U*-test was used to determine the significance among multiple comparisons. All data were analyzed by one-way ANOVA unless mentioned. In the water maze test, escape latency was analyzed using a repeated one-way ANOVA followed by Fisher’s least significant difference test. *P* < 0.05 was considered as statistical significance.

## Results

### NP65 deletion attenuates cognitive deficits in AD mice

Firstly, the offspring genotypes of NP65^+/–^APP/PS1 male mice with NP65^+/–^ female mice were identified by PCR using tail DNA extracts at the age of 4 weeks. The six genotypes of the offspring were identified (shown in Supplementary Figure 1). After PCR identification, these genotypes of WT, KO, AD, and KOAD mice were bred until use. The gross macroscopic features of KOAD mice were observed, and we did not find the differences between KO and KOAD mice, including body weight, appetite, drinking water, temperature and gross brain size. In addition, NP65 deletion in AD mice at the age of 2 months was further confirmed by western blot using NP65 antibody. No NP65 expressions were detected in forebrains from KO and KOAD mice, while similar levels were detected in forebrains from WT and AD mice (Supplementary Figure 2).

**FIGURE 1:**
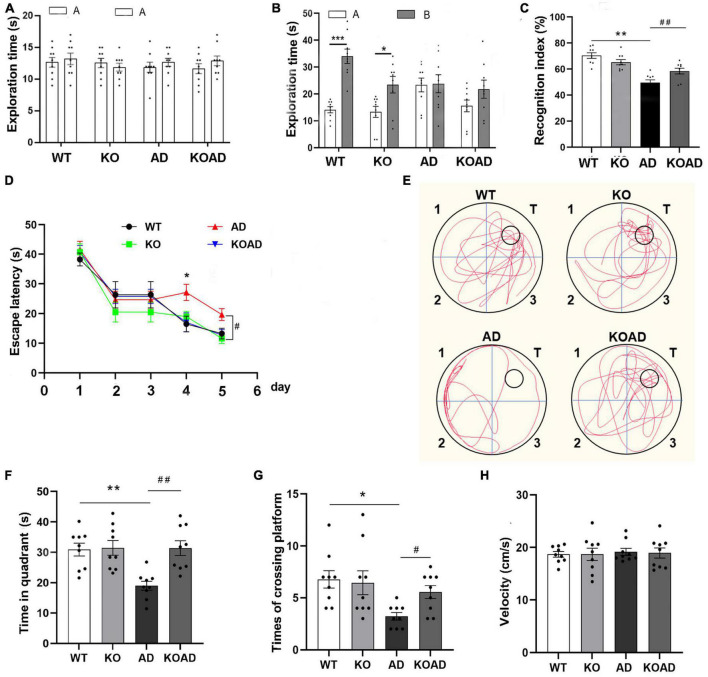
Neuroplastin 65 (NP65) deficiency attenuates cognitive deficits in AD mice. In a novel object recognition test, for exploration time in two identical objects **(A)** or in the familiar and novel objects **(B)**, KOAD mice showed a higher recognition index [RI, **(C)**] compared with AD mice. **(D–H)** In the Morris water maze (MWM) assay, the learning curve of mice on five consecutive days **(D)**. In the probe test, representative runs in the probe trials were shown **(E)**. KOAD mice spent more time in the target **(F)** and took more entries of crossing platform compared with AD mice **(G)**. **(H)** On day 7, during the visible probe test, swimming speed did not differ between any groups. AD vs. WT and KO, *P < 0.05, **P < 0.01, ***P < 0.001; KOAD vs. AD, ^#^*P* < 0.05, ^##^*P* < 0.01.

Secondly, the NOR and MWM tests were used to determine whether NP65 deletion is associated with cognitive behaviors of AD mice at the age of 10 months, when obvious cognitive deficits are observed in this AD mouse model. In the NOR test, mice from all groups displayed a comparable exploration time for two identical objects (Figure 1A), while during exploration of novel and familiar objects (Figures 1B, C), AD mice showed a significantly lower RI compared with WT and KO mice, consistent with our previous reports (Li et al., 2019). Notably, KOAD mice significantly reversed the lower RI compared with AD mice (Figures 1B, C), suggesting that NP65 deficiency alleviates spatial deficits in this AD mouse model. In addition, during spatial learning course in the MWM test, these mice spent similar time to find the submerged platform on day 1–3 of the 5 consecutive days; while AD mice spent longer time to find this platform on day 4–5 compared with WT and KO mice (Figure 1D). During the probe test (platform removed, Figures 1E–G), AD mice spent less time in the target quadrant than WT and KO mice; but KOAD mice spent more time in the target and took more entries of crossing platform compared with AD mice (Figures 1F, G). In visible probe test, swimming speeds did not differ among these groups (Figure 1H). These results indicate that NP65 deficiency alleviates spatial deficits in AD mice.

### NP65 deficiency causes a decrease in both Aβ levels and Aβ deposition in AD mouse brains

**FIGURE 2:**
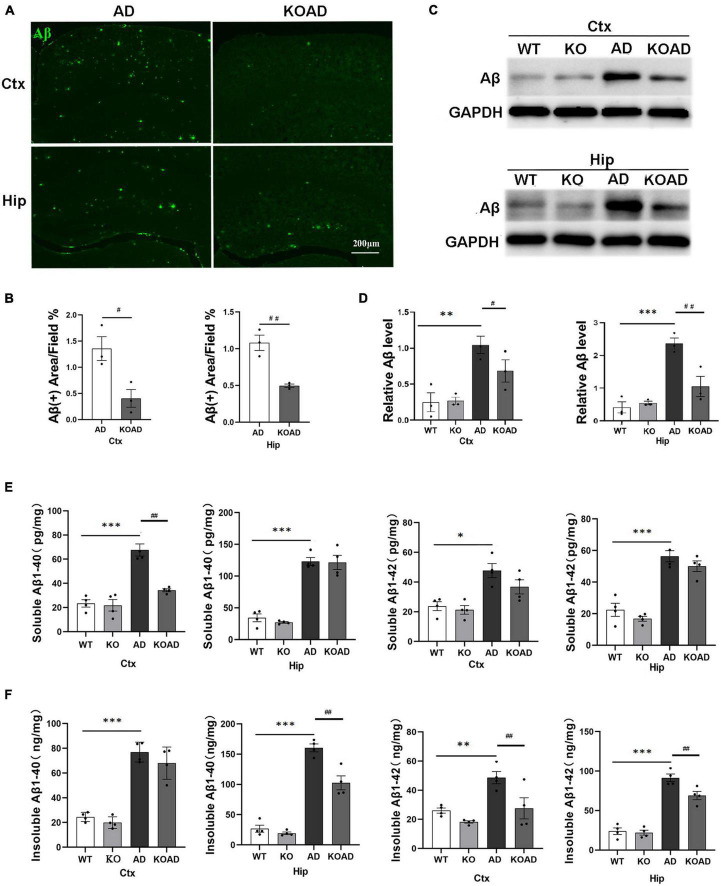
Neuroplastin 65 (NP65) deficiency results in a decrease in both Aβ levels and Aβ deposition in the brain of AD mice. Immunofluorescent staining using 6E10 antibody showed Aβ plaques in the cortex (Ctx) and hippocampus (Hip) in AD and KOAD mice **(A)**, and quantification **(B)** revealed a remarkable reduction in Aβ occupied area in KOAD mice. Western blots showed representative immunoblot bands of Aβ in the cortex and hippocampus **(C)**, and the quantification **(D)** revealed Aβ levels in KOAD mice were significantly reduced compared with AD mice, whereas WT and KO mice showed lower similar Aβ levels. **(E,F)** ELISA documented that AD mice showed a strong increase in insoluble/soluble Aβ1–40 and Aβ1–42 in the cortex and hippocampus compared with WT and KO mice, but KOAD mice displayed a remarkable reduction in insoluble Aβ1–42 levels of the cortex and hippocampus and insoluble Aβ1–40 levels of hippocamps and soluble Aβ1–40 levels in the cortex compared with AD mice. AD vs. WT and KO, *P < 0.05, ^**^P < 0.01, ^***^P < 0.001; KOAD vs. AD, ^#^*P* < 0.05, ^##^*P* < 0.01.

Given that APP/PS1 mice exhibit detectable Aβ plaques at the age of 6 months (Liu et al., 2020), 10-month-old AD and KOAD mice were examined to determine whether NP65 deficiency would affect Aβ levels and Aβ plaques in AD mice. Using immunostaining, a broad distribution of Aβ plaques was found throughout the cortex and hippocampus in AD and KOAD mice (Figure 2A), but KOAD mice showed a significant reduction in Aβ burden (6E10-positive) area in the cortex and hippocampus (Figure 2B). In addition, western blot analysis revealed that KOAD mice displayed a significant decrease in Aβ levels of the cortex and hippocampus compared with AD mice (Figures 2C, D). Finally, the levels of Aβ1–40 and Aβ1–42, the main components of Aβ peptides, were measured by ELISA. As expected, AD mice exhibited a strong increase in insoluble/soluble Aβ (1–40 and 1–42) levels compared with WT and KO mice (Figures 2E, F). Meanwhile, WT and KO mice had comparable levels of insoluble and soluble Aβ1–40 and Aβ1–42 in the cortex and hippocampus (Figures 2E, F). Interestingly, KOAD mice displayed a significant decrease in insoluble Aβ1–42 levels in the cortex and hippocampus, insoluble Aβ1–40 levels in the hippocampus and soluble Aβ1–40 in the cortex compared with AD mice (Figures 2E, F). Taken together, these results demonstrate that NP65 deficiency results in a decrease in both Aβ levels and Aβ deposition in the APP/PS1 mouse model of AD.

### NP65 deficiency does not affect BACE1 expression in AD mice

**FIGURE 3:**
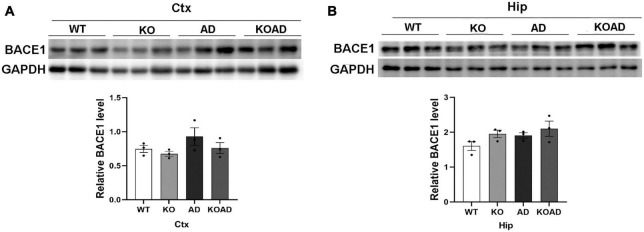
Neuroplastin 65 (NP65) deficiency does not alter BACE1 protein levels in AD mice. Representative BACE1 immunoreactive bands in the cortex [Ctx, **(A)**] and hippocampus [Hip, **(B)**] from WT, KO, AD, and KOAD mice, and quantification [the lower panels of **(A,B)**] showed no statistical difference among these genotypes. Data are shown as mean ± SEM from three independent experiments and were analyzed Mann-Whitney *U*-test. BACE1, β-site APP cleaving enzyme 1; GAPDH, Glyceraldehyde-3-phosphate Dehydrogenase.

As known, NP65 is located on pre-synaptic and post-synaptic membranes (Herrera-Molina et al., 2014). BACE1 is a crucial proteolytic enzyme to produce Aβ peptide and highly expressed on the dystrophic neurites in AD mice (Zhang et al., 2009). To examine whether NP65 deficiency affects BACE1 levels as a possible cause of reduced Aβ plaques, we determined BACE1 levels by western blot and found no difference in BACE1 protein levels in the hippocampus and cortex among different genotypes (Figures 3A, B). These results suggest that NP65 deficiency has no effect on BACE1 expression in AD mice.

### NP65 deficiency leads to less activation of microglia and has no effect on microglial Aβ phagocytosis in AD mice

**FIGURE 4:**
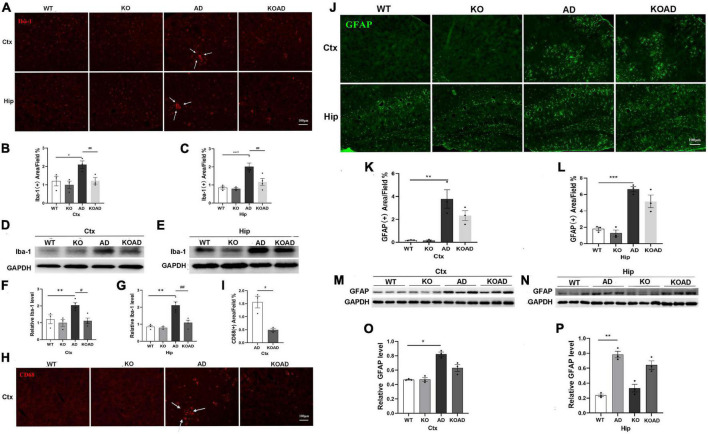
Neuroplastin 65 (NP65) deficiency has effect on glial activation in AD mice. Representative Iba-1(+) microglia **(A)** in the cortex and hippocampus among different genotypes, and clustered Iba-1(+) microglia [arrows in **(A)**] in AD and KOAD mice; quantification **(B,C)** of Iba-1(+) microglial area in KOAD mice showed a significant decrease compared with AD mice. Representative Iba-1 immunoreactive bands **(D,E)** and quantification **(F,G)** indicating a significant reduction in Iba-1 protein level in KOAD mice relative to AD mice. Representative CD68(+) microglia **(H)**, clustered CD68(+) microglia [indicated in arrows in **(H)**], and quantification **(I)** of CD68(+) area in KOAD mice displaying a remarkable decrease compared with AD mice. Representative GFAP(+) astrocytes **(J)** in the cortex and hippocampus among different genotypes, and quantification **(K,L)** showing no difference between KOAD and AD mice. Representative GFAP immunoreactive bands **(M,N)** and quantification **(O,P)** confirmed no statistical difference in GFAP protein levels between KOAD and AD mice. AD vs. WT and KO, *P < 0.05, **P < 0.01, ***P < 0.001; KOAD vs. AD, ^#^*P* < 0.05, ^##^*P* < 0.01.

**FIGURE 5:**
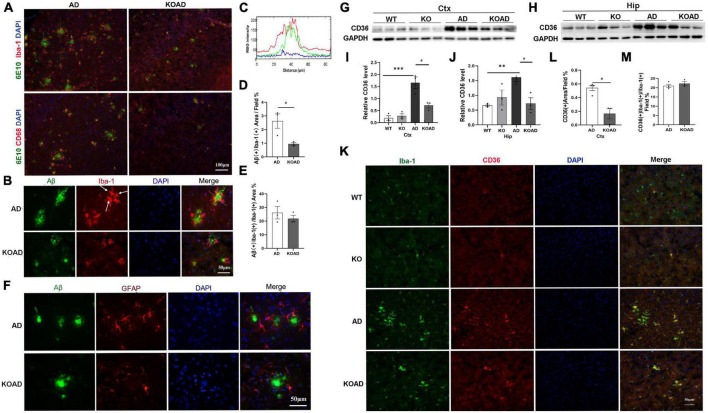
Neuroplastin 65 (NP65) deficiency exhibits no effect on glial Aβ phagocytosis in AD mice. Representative double-immunostaining of 6E10/Iba-1 and 6E10/CD68 in the cortex from AD and KOAD mice, intracellular Aβ [yellow color in **(A,B)**] inside microglia, nuclei stained by DAPI (blue). **(C)** RGB intensity profile of Aβ (green line) and Iba-1 (red line) are shown according to the corresponding RGB image of area (simplified dimensioning line, distance 0–1 = 80 μm) in **(B)** (right upper pane). RGB intensity profiling localizes peak Aβ (green) intermediate to peak Iba-1 (red) fluorescence intensity. Quantification of microglial Aβ phagocytosis, showing a significant decrease in intracellular Aβ levels (Aβ + /Iba-1 +) in KOAD mice compared to AD mice **(D)**, while the percentage of intracellular Aβ area showed no difference between KOAD and AD mice **(E)**. **(F)** Representative double-immunostaining of the GFAP (red) with 6E10 (Aβ, green) in the cortex from AD and KOAD mice and nuclei stained by DAPI (blue), no colocalization of the 6E10/GFAP was observed. Representative CD36 immunoreactive bands of the cortex and hippocampus **(G,H)** and quantification **(I,J)** showed that CD36 levels in KOAD mice were significantly decreased compared with AD mice while no difference was observed between WT and KO mice. Double immunostaining of the Iba-1/CD36 in the cortex **(K)** from WT, KO, AD, and KOAD mice, nuclei stained by DAPI (blue), and quantification of CD36 levels **(L)** and percentage of CD36 + microglia **(M)** between KOAD and AD mice. AD vs. WT and KO, ^**^P < 0.01, ^***^P < 0.001; KOAD vs. AD, ^#^*P* < 0.05.

Next, we observed glial activation to determine whether NP65 deficiency affects glial response in AD mice. As shown in Figure 4, immunostaining with Iba-1 antibody showed that microglia in WT and KO mice were evenly distributed throughout the cortex and hippocampus without difference. However, a few microglia in AD and KOAD mice were clustered (arrows in Figures 4A, 5B), as reported previously in AD mice (Liu et al., 2020). Quantification of Iba-1(+) microglial area in the cortex and hippocampus revealed a significant decrease in KOAD mice relative to AD mice, whereas no difference was observed between KO and WT mice (Figures 4B, C). Additionally, western blot analysis showed the decreased Iba-1 protein levels in KOAD mice compared with AD mice, while no evident change was found between KO and WT mice (Figures 4D–G). Furthermore, we measured the expression level of CD68, a phagocytic marker for microglia. The result showed that there was a strong reduction of CD68 (+) area in the cortex of KOAD mice compared with AD mice (Figures 4H, I). As expected, very few CD68 (+) microglia were detected in WT and KO mice (Figure 4I). Together, these results suggest that NP65 deficiency causes less microglial activation in AD mice.

In addition, we also explored the activation of astrocytes using GFAP antibody. As shown in Figure 4J, GFAP (+) astrocytes were located throughout cortex and hippocampus between WT and KO mice. The numbers of GFAP (+) astrocytes in AD mice were significantly increased compared with WT mice, but were comparable to those compared with KOAD mice (Figures 4J–L). Consistent with immunostaining results, KOAD mice showed similar GFAP levels compared with AD mice by western blots; and no difference was observed between WT and KO mice as well (Figures 4M–P). Together, these results suggest that NP65 deficiency has no effect on the astrocytic activation in AD mice.

Finally, Aβ phagocytosis indicated by intracellular 6E10 immunostaining inside the microglia and astrocytes, was investigated using double-immunostaining for 6E10 with Iba-1, CD68 or GFAP as previously reported (Pan et al., 2019; Liu et al., 2020). The merged images of 6E10/CD68 or 6E10/Iba-1 microglia clearly showed intracellular Aβ (indicated by yellow color in Figures 5A, B) inside microglia. Using a RGB fluorescence intensity profile as previously described (Krauthausen et al., 2015), we observed the intracellular Aβ inside microglia (Figure 5C). Quantitative analysis showed that intracellular Aβ levels inside Iba-1 (+) microglia in KOAD mice were significantly decreased compared with AD mice (Figure 5D). However, the percentage of intracellular Aβ area in microglia showed no significant difference between KOAD and AD mice (Figure 5E), suggesting the comparable microglial capacity for Aβ phagocytosis. In contrast, no colocalization of the 6E10/GFAP was observed in AD and KOAD mice (Figure 5F). These results show that NP65 deficiency has no effect on Aβ phagocytic capacity of microglia in AD mice.

Scavenger receptor CD36, mainly expressed in microglia, is known to mediate Aβ phagocytosis of microglia. To further investigate the Aβ phagocytosis of microglia, we examined CD36 expression in NP65-deficient AD mice. Western blot analysis showed that KOAD mice exhibited a significant decrease of CD36 level in the cortex and hippocampus compared with AD mice. Consistent with previous reports (Liu et al., 2020), AD mice had significantly higher CD36 levels than WT and KO mice (Figures 5G–J). These results were further confirmed by double-immunostaining for Iba-1/CD36 showing that CD36 was exclusively expressed in microglia and the CD36 (+) area were apparently decreased in KOAD mice compared to AD mice (Figures 5K, L). Notably, the percentage of CD36 (+) microglia was comparable between KOAD and AD mice (Figure 5M). Therefore, the decreased CD36 levels may result from the reduced amounts of microglia in KOAD mice.

### NP65 deficiency exhibits no effect on microglial phenotype in AD mice

**FIGURE 6:**
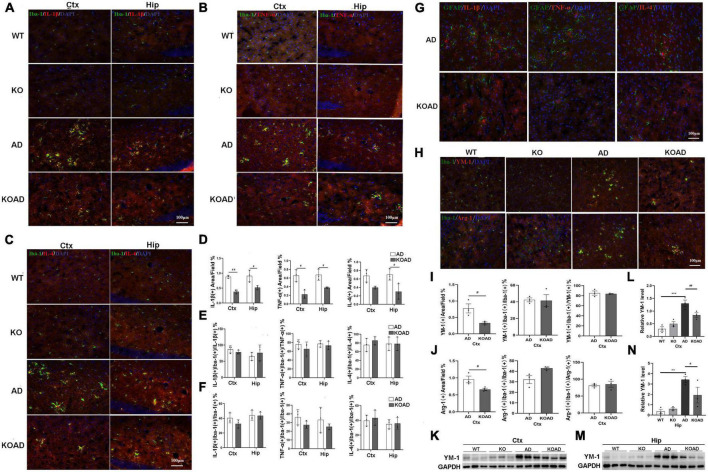
Neuroplastin 65 (NP65) deficiency exhibits no effect on microglial phenotype in AD mice. Representative double-immunostaining of Iba-1/IL-1β **(A)**, Iba-1/TNF-α **(B)**, and Iba-1/IL-4 **(C)** in WT, KO, AD, and KOAD mice, nuclei stained by DAPI (blue). Quantification of immunostaining for Iba-1 with TNF-α, IL-1β, and IL-4 demonstrated a significant reduction in IL-1β, TNF-α, and IL-4 levels in KOAD mice compared with AD mice **(D)** while the ratio of co-localization to total TNF-α, IL-1β, or IL-4 levels **(E)** and the percentage of microglia expressing TNF-α, IL-1β, or IL-4 **(F)** was comparable between KOAD and AD mice. **(G)** Representative double-immunostaining of GFAP with TNF-α, IL-1β, or IL-4 in the cortex of AD and KOAD mice and nuclei stained by DAPI (blue). No double-positive astrocytes were detected. Double-immunostaining of Iba-1 with YM-1 and Arg-1 in the cortex and hippocampus **(H)** and nuclei stained by DAPI (blue). Quantitative analysis **(I,J)** shows a significant decrease of YM-1(+) or Arg-1(+) area in KOAD mice compared with AD mice while the percentage of microglia expressing YM-1 or Arg-1 and the ratio of co-localization to YM-1 or Arg-1 were comparable between KOAD and AD mice **(I,J)**. Representative immunoreactive bands **(K,M)** of YM-1 in WT, KO, AD, and KOAD mice and quantitative analysis **(L,N)** exhibiting a significant decrease in levels of YM-1 of hippocampus and cortex in KOAD mice than AD mice. AD vs. WT and KO **P < 0.01, ***P < 0.001; KOAD vs. AD, ^#^*P* < 0.05, ^##^*P* < 0.01. IL-1β, interleukin-1beta; GAPDH, glyceraldehyde-3-phosphate dehydrogenase; TNF-α, tumor necrosis factor alpha; IL-4, interleukin-4; YM-1, chitinase 3-like 3; Arg1, Arginase 1; DAPI, diamidinyl phenyl indole.

After exposing to amyloid plaques, activated glia produce pro-inflammatory and anti-inflammatory cytokines to propel the pathological processes in AD. Thus, the pro-inflammatory and anti-inflammatory cytokines were examined to explore whether NP65 deficiency affects glial response in AD mice. Pro-inflammatory factors including TNF-α and IL-1β, and anti-inflammatory cytokine IL-4 were examined using double-immunostaining. The co-localization of Iba-1/TNF-α, Iba-1/IL-1β, and Iba-1/IL-4 confirmed that TNF-α, IL-1β, and IL-4 were mainly expressed in clustered microglia in AD and KOAD mice, suggesting that the majority of TNF-α, IL-1β, and IL-4 are produced by microglia. In contrast, few expressions were observed in WT and KO mice (Figures 6A–C). Quantification results demonstrated a significant reduction in IL-1β, TNF-α, and IL-4 levels of hippocampus and cortex in KOAD mice compared with AD mice (Figure 6D). However, the ratio of co-localization to total TNF-α, IL-1β, or IL-4 levels was not changed between KOAD and AD mice (Figure 6E), suggesting that microglia are still the major source for these inflammatory factors in KOAD mice. Furthermore, the percentage of microglia expressing TNF-α, IL-1β, or IL-4 was comparable between KOAD and AD mice (Figure 6F), indicating that NP65 deficiency has no effect on microglial production of TNF-α, IL-1β, and IL-4. These results show that proportion of M1 classic (producing pro-inflammatory cytokines) and M2 alternative (producing anti-inflammatory cytokines) phenotype of microglia is unaltered in NP65-deficient AD mice. In addition, double-immunostaining for GFAP/TNF-α, GFAP/IL-1β, and GFAP/IL-4 was performed and showed no detectable double positive-astrocytes in the cortex of AD and KOAD mice (Figure 6G).

As protective matrix YM-1 and Arg-1 also serve as the markers for M2 phenotype of microglia, the expressions of YM-1 and Arg-1 were then determined by immunostaining. As shown in Figure 6H, very few YM-1(+) or Arg-1(+) staining was detected in the cortex of WT and KO mice, while many YM-1(+) and Arg-1(+) areas were present in AD mice. Notably, KOAD mice had smaller YM-1(+) and Arg-1(+) areas than AD mice, while the percentage of YM-1(+) or Arg-1(+) microglia was similar between AD and KOAD mice (Figures 6I, J), suggesting that NP65 deficiency does not affect microglial production of YM-1 and Arg-1. Moreover, western blot analysis also confirmed that KOAD mice displayed a significant decrease in levels of YM-1 of the hippocampus and cortex compared with AD mice (Figures 6K–N). Taken together, these results indicate that NP65 deficiency has no effect on microglial phenotype in AD mice.

### NP65 deficiency results in the decreased expressions of Htr3A in AD mice

**FIGURE 7:**
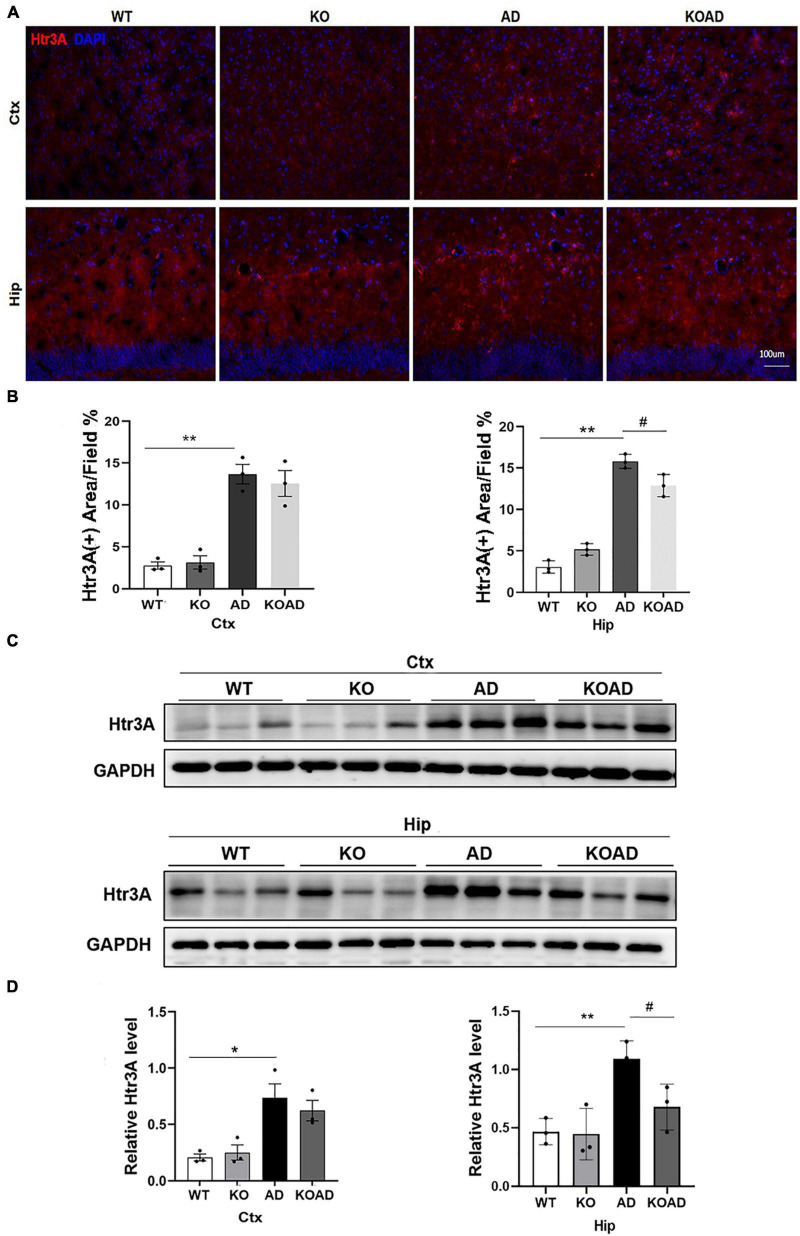
Neuroplastin 65 (NP65) deficiency causes the reduction of Htr3A protein levels in AD mice. Representative Htr3A immunostaining **(A)** in the cortex (Ctx) and hippocampus (Hip) of all genotypes, nuclei stained by DAPI (blue), and quantitative analysis **(B)** confirmed that KOAD mice displayed a significant decrease in Htr3A expressions of hippocampus compared with AD mice. Representative immunoreactive bands **(C)** of Htr3A in WT, KO, AD, and KOAD mice. Quantitative analysis **(D)** exhibited a significant increase in Htr3A levels in the hippocampus and cortex of AD mice relative to WT mice, while NP65 deletion led to a significant decrease in Htr3A levels of the hippocampus of AD mice. AD vs. WT or KO, *P < 0.05, **P < 0.01; KOAD vs. AD, ^#^*P* < 0.05.

Our previous study has confirmed that Htr3A expressions were significantly increased and inhibiting Htr3A expressions decreased Aβ plaques in brains of this AD mouse model (Liu et al., 2023). For Htr3A is expressed in specific subtypes of interneurons in the hippocampus and cortex, where NP65 is highly expressed. Thus, we argue whether NP65 deficiency could affect Htr3A expressions to mitigate Aβ plaques in AD mice. Immunostaining showed that Htr3A expressions in AD mice was significantly increased compared with WT mice, which is consistent with our previous results (Liu et al., 2023). Notably, KOAD mice showed a remarkable decrease in Htr3A expressions of the hippocampus compared with AD mice (Figures 7A, B). Additionally, western blot analysis also confirmed that KOAD mice displayed a significant decrease in Htr3A levels of the hippocampus in relative with AD mice (Figures 7C, D). Collectively, these findings suggest that the decreased Aβ plaques in NP65-deficient AD mice may be correlated with the decreased Htr3A expressions.

### NP65 deficiency does not alter synaptic, NeuN, and Bax protein levels in AD mice

**FIGURE 8:**
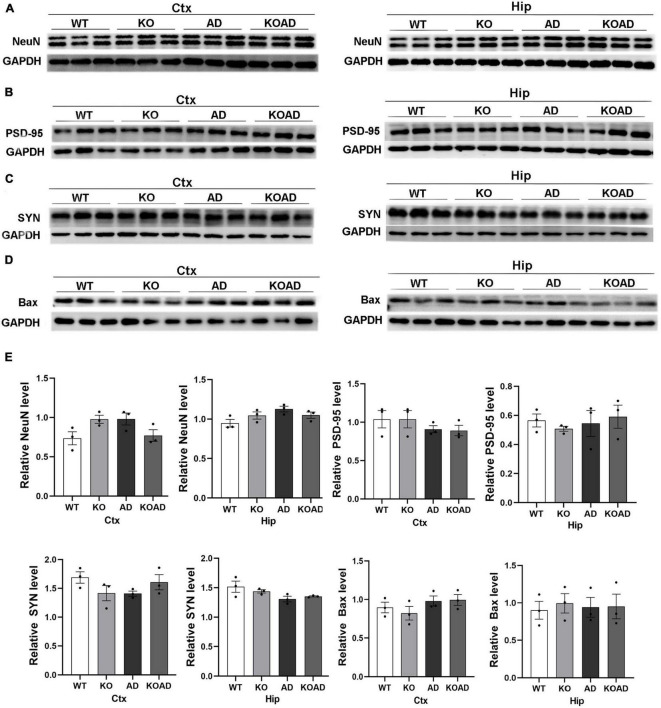
Neuroplastin 65 (NP65) deficiency has no influence in neuron and synaptic protein expression in AD mice. Representative immunoreactive bands for NeuN **(A)**, post-synaptic density (PSD-95) **(B)**, synaptophysin (SYN) **(C)**, and apoptotic protein Bax **(D)** in the hippocampus and cortex and quantification **(E)** showing no significant difference among different genotypes at the age of 10 months. Data were collected from three independent experiments.

The loss of synaptic proteins and neurons is one of the major pathological changes in AD. Therefore, we wondered whether NP65 deficiency affects the expression of neuronal and synaptic proteins in AD mice at the age of 10 months. The levels of NeuN (neuronal marker), and synaptic proteins including SYN and PSD95 and apoptotic protein Bax were determined. Immunoblot analysis showed that the levels of NeuN, PSD95, SYN, and Bax were not significantly different in the cortex and hippocampus among these groups (Figures 8A–E). These results suggest that NP65 deficiency has no influence on neuronal and synaptic protein expression in AD mice at the age of 10 months.

## Discussion

Growing studies show that NP65 correlates with learning and memory in rodents (Ilic et al., 2021; Lin et al., 2021). However, no report provides the direct evidence for the role of NP65 in cognitive disease, especially in AD dementia. Our study provides the first direct evidence that NP65 is implicated in cognitive deficits of AD mouse model. Using the NP65-deficient AD mouse model, we found that NP65 deficiency alleviates cognitive deficits and leads to a decrease in Aβ levels and Aβ plaques in APP/PS1 mice.

Alzheimer’s disease is clinically characterized as progressive memory impairment, and whether specific molecules are implicated in this course remains elusive. As our previous study showed an enhanced spatial memory in NP65-deficient mice (Amuti et al., 2016; Li et al., 2019), Thus, we wondered whether NP65 deficiency could attenuate the cognitive deficits in APP/PS1 mice. As expected, NP65-deficient APP/PS1 mice performed better in NOR and MWM tests compared with control, suggesting that NP65 is involved in cognitive deficits in APP/PS1 mouse model of AD. Coincidently, a study reported an alteration of NP65 expression in the hippocampus of AD patients at different phases (Ilic et al., 2019). Collectively, our findings directly indicate the role of NP65 in the cognitive deficits of APP/PS1 mice.

Amyloid plaques, the main pathological hallmark of AD, are composed of extracellular Aβ deposition and dystrophic neuritis and mainly located in the hippocampus and cerebral cortex at the early stage of AD (Fiala, 2007). Aβ plaques are well considered to play important roles in AD pathogenesis by causing glial activation, neuroinflammation and loss of neurons and synapses. Thus, Aβ levels and Aβ burden were measured in NP65-deficient AD mice. Interestingly, KOAD mice displayed a significant decrease in total Aβ levels and plaque burden of the hippocampus and cortex than AD mice determined by western blot and immunostaining. Furthermore, ELISA analysis showed that KOAD mice had a significant decrease in insoluble Aβ1–42 levels of the hippocampus and cortex, and insoluble Aβ1–40 levels in hippocampus compared with AD mice. Given the characteristics of NP65 as a cell adhesion molecule and NP65 located pre-synaptic and post-synaptic terminals, it is inferred that the extracellular Ig module of NP65 could bind to Aβ peptides especially Aβ1–42 to promote Aβ aggregation resulting in Aβ plaques. Consistently, NP65 has been shown to interact with the amino-terminal of GluA1 in AMPA receptors and α1 or α2 subunits in GABA (A) receptors to modify synaptic transmission (Sarto-Jackson et al., 2012; Jiang et al., 2021). However, our speculation needs to be demonstrated by using Co-IP and double-immunostaining to show the interaction NP65 with Aβ1–42 and Aβ1–40 in our next research. In addition, another interesting finding is that KOAD mice showed a remarkable decrease in soluble Aβ1–40 levels of cortex compared with AD mice, suggesting the reduction of the Aβ generation in the cortex. Taken together, the present findings provide direct evidence that NP65 is correlated with the generation and aggregation of Aβ in APP/PS1 mice.

Aβ plaques at the early stage of AD are localized in sublayers of the hippocampus where interneurons are located (Lee et al., 2010; Krauthausen et al., 2015; Pan et al., 2019; Liu et al., 2020). Our previous study showed that Htr3A interneurons partially contribute to Aβ generation and inhibiting Htr3A expressions reduces Aβ plaque levels in this AD mice (Liu et al., 2023). For NP65 is highly expressed in hippocampus (Smalla et al., 2000; Herrera-Molina et al., 2014), we wanted to know whether NP65 deficiency would affect Htr3A interneurons and then to reduce Aβ plaque levels in this AD mice. Consistent with our previous results, AD mice displayed a significant increase in the Htr3A expressions compared with WT mice (Liu et al., 2023). Interestingly, immunostaining and western blot analysis confirmed that NP65 deficiency partially decreased Htr3A expressions in hippocampus of AD mice. Therefore, the decreased Aβ levels in hippocampus of NP65-deficient AD mice may be related with the decreased Htr3A expressions. Recent studies have revealed that NP65/55 is essential for expression of plasma membrane Ca (2^+^) ATPase (PMCA) and essential auxiliary subunits of PMCA and key regulators of Ca^2+^ clearance (Bhattacharya et al., 2017; Herrera-Molina et al., 2017; Schmidt et al., 2017; Gong et al., 2018; Newton et al., 2022). Ca^2+^ dys-homeostasis could promote the accumulation of Aβ and brain function deficits in AD patients (Mark et al., 1995; Khachaturian, 2017). Given that Htr3 is an ionotropic receptor with permeability to Ca^2+^ (Maricq et al., 1991), it is possibly that NP65 could change Ca^2+^ dys-homeostasis in Htr3A interneurons contributing to Aβ generation. For NP65 is located in presynaptic terminals and Aβ generation comes largely from APP- and BACE1-positive presynaptic terminals in AD mice (Zhang et al., 2009), it is speculated that NP65 interacts with specific proteins related with APP processing in presynaptic terminals to mediate Aβ level. Using Co-IP and Protein mass spectrometry to screen out and identify interacted proteins with NP65 is the way to find underlying mechanisms that NP65 affects Aβ generation in AD mice in our next research.

To uncover how NP65 deficiency decreases Aβ levels in AD mice, Aβ phagocytosis of microglia and inflammatory response were explored in NP65-deficient AD mice. Although NP65 deficiency resulted in the decreased amounts of activated microglia in AD mice, the capacity of Aβ phagocytosis of microglia was unaltered between KOAD and AD mice. In parallel with reduced microglial amounts, we observed that inflammatory cytokines (TNF-α, IL-1β, and IL-4) and protective matrix (YM-1 and Arg-1) were remarkably decreased in NP65-deficient AD mice. However, the percentage of microglia expressing these cytokines and matrix showed no difference between KOAD and AD mice, suggesting that the proportion of M1 classic and M2 alternative phenotype of microglia is unchanged in NP65-deficient AD mice. Except microglial source, these cytokines (TNF-α, IL-1β, and IL-4) and matrix YM-1 and Arg-1 can be produced by neurons and astrocytes (Liu et al., 2020; Zhang et al., 2021). In our results, KOAD and AD mice displayed no differences in the ratio of Iba-1(+) cytokines (TNF-α, IL-1β, and IL-4) and matrix (YM-1 and Arg-1) to total these cytokines and matrix levels, indicating similar cellular sources of these cytokines and matrix between KOAD and AD mice. Taken together, our results confirmed that NP65 deficiency has no effect on microglial phenotype and neuroinflammation in this AD mouse model. Thus, the decreased levels of Aβ plaques result in less microglial activation and ensuing reduced inflammatory cytokines in KOAD mice.

In conclusion, we have presented evidence that NP65 deficiency alleviates cognitive deficits and decreases Aβ levels and Aβ plaques in APP/PS1 mice. However, the mechanisms underlying NP65 affects Aβ plaque levels need to be further explored in future study.

## Data availability statement

The raw data supporting the conclusions of this article will be made available by the authors, without undue reservation.

## Ethics statement

This animal study was reviewed and approved by the Tongji University Animal Care Committee.

## Author contributions

Q-LY designed the experiments and wrote the manuscript. D-DW, Y-TL, JC, Y-NZ, and L-FL performed the experiments. S-XH and LH analyzed the data. All authors contributed to the article and approved the submitted version.

## Supplementary material

Supplementary Figure 1 The offspring genotypes (WT, KO, AD, and KOAD) of NP65^+/–^APP/PS1 male mice with NP65^+/–^ female mice are performed by PCR of tail DNA extracts.

Supplementary Figure 2 Neuroplastin 65 (NP65) deletion in AD mice was further confirmed by western blot using NP65 antibody.
