# Supplementary figures and images for: Neuroplastin 65 deficiency reduces amyloid plaque formation and cognitive deficits in an Alzheimer’s disease mouse model
Source: Front Cell Neurosci. 2023 May 5;17:1129773. doi: 10.3389/fncel.2023.1129773 (PMC10196121; doi:10.3389/fncel.2023.1129773)

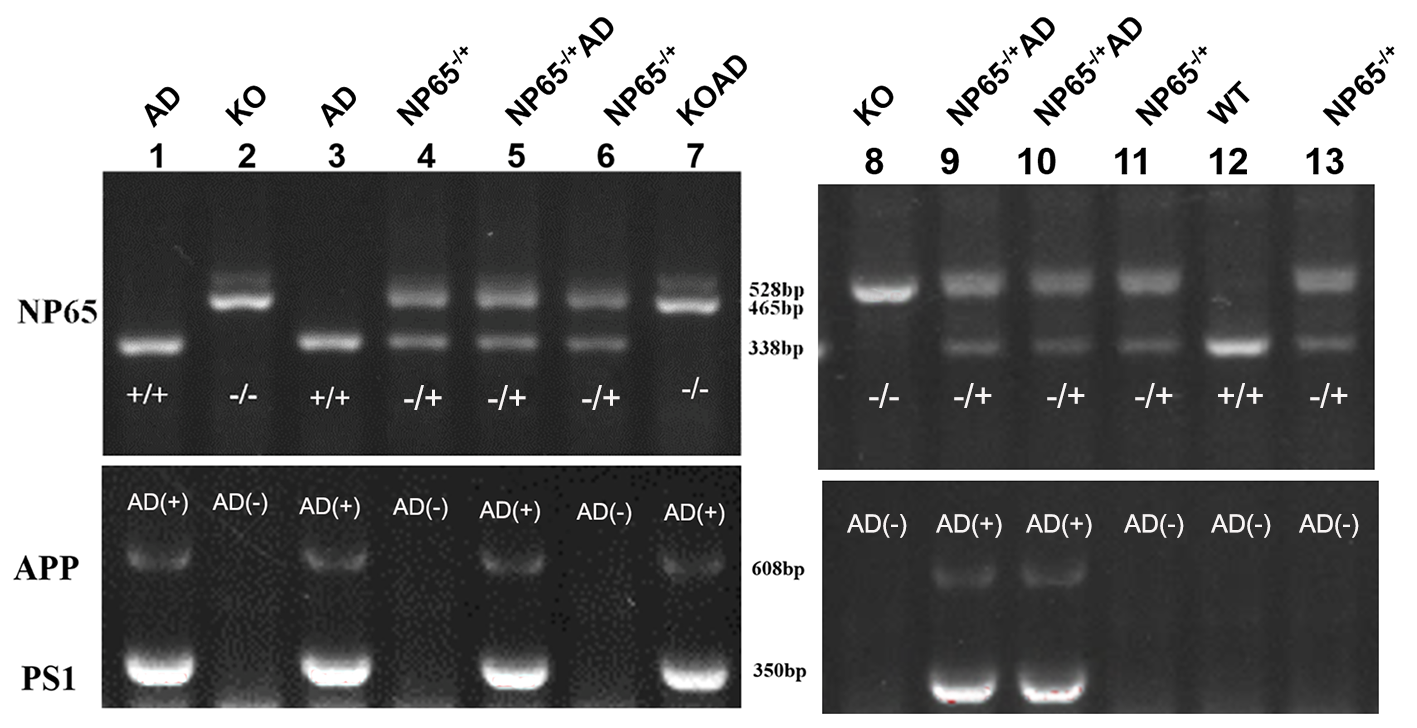

Supplement: Supplementary Figure 1 — The offspring genotypes (WT, KO, AD, and KOAD) of NP65+/–APP/PS1 male mice with NP65+/– female mice are performed by PCR of tail DNA extracts. [file Image_1.TIF]

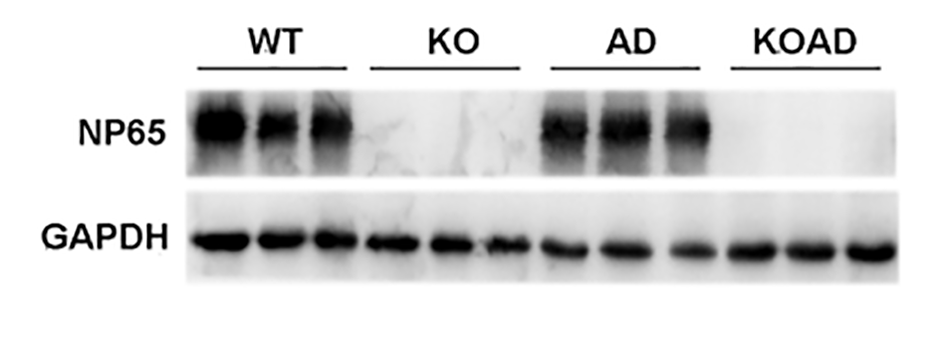

Supplement: Supplementary Figure 2 — Neuroplastin 65 (NP65) deletion in AD mice was further confirmed by western blot using NP65 antibody. [file Image_2.TIF]
